# Assessment of Intestinal Parasites and Its Associated Factors among Fruits and Vegetables Collected from Local Markets of Bule Hora Town, Southeast Ethiopia

**DOI:** 10.1155/2023/1861919

**Published:** 2023-09-21

**Authors:** Tibeso Gemechu, Jemal Bona, Alqeer Aliyo, Wako Dedecha, Girma Ashenafi

**Affiliations:** Department of Medical Laboratory Science, College of Health Sciences, Bule Hora University, Bule Hora, Ethiopia

## Abstract

**Background:**

Vegetable and fruit consumptions are important for health as they are good sources of carbohydrates, vitamins, minerals, and fiber. However, contamination of vegetables and fruits is indicated as the main contributing factor to parasitic contamination.

**Objective:**

This study aims to assess the prevalence and associated factors of intestinal parasites among fruits and vegetables collected from local markets in Bule Hora Town, Southeast Ethiopia.

**Methods:**

A cross-sectional assessment was carried out on 391 raw fruits and vegetables from the market in Bule Hora Town from July 29 to August 17, 2022. After being soaked in physiological saline and vigorously shaken for 15 minutes with the help of a mechanical shaker, a total of 391 samples taken from various fruits and vegetables were evaluated using the sedimentation concentration technique. Software SPSS version 25 was used to analyze the data after it had been entered using EpiData version 3.1. To assess various associated factors, binary and multivariable logistic regression was employed.

**Results:**

142 (36.3%) of the 391 samples analyzed had at least one species of parasite. The parasite *Ascaris lumbricoides* (40.1%) was found the most frequently, whereas *Strongyloides* spp. was found the least frequently. Variables such as fingernail trimming (AOR = 1.99; 95% CI: 1.274–3.108), hand washing habit with soap after using toilet (AOR = 2.912; CI: 1.896–4.47), and eating raw vegetables or fruits (AOR = 0.604; CI: 0.394–0.925) were associated with parasitic contamination.

**Conclusions:**

The results of this study show that eating vegetables that are raw in the study area carries a potentially significant risk of contracting parasitic illnesses. Therefore, the appropriate bodies should make an effort to lower the rate of product contamination with intestinal parasites by educating vendors and the general public.

## 1. Introduction

Intestinal parasite disease is one of the infectious diseases caused by species of protozoa, cestodes, trematodes, or nematodes. In impoverished nations, these parasites are mostly to blame for illness and mortality [1, 2]. Due to poor environmental cleanliness, personal hygiene practices, and general health system weaknesses, intestinal contaminations are very common in underdeveloped nations [3–5]. About 3.5 billion people are affected globally, of which 450 million suffer from intestinal parasite diseases, which are thought to be the cause of 200,000 annual deaths [6]. As a significant part of a balanced diet, fruits and vegetables are excellent for maintaining health and preventing disease, but they also have the potential to spread several infectious and parasitic disorders [7]. Fruits and vegetables can become infected with parasites in their infective stages, and the occurrence of parasite ova, cysts, and larva on fruits and vegetables depends on contamination with untreated wastewater, poor personal hygiene, and the use of human feces as fertilizer on farms [8].

Most fruits and vegetables are consumed raw or very gently cooked to maintain their natural flavor and nutrients that are sensitive to heat [1, 2]. However, this practice encourages the spread of food-borne illnesses. In preharvest (cultivation, irrigation, and livestock manure) and postharvest handling-storage or transit or during processing for consumption, fruits and vegetables may be exposed to various parasitic stage pollutants[1, 3].

Most local farmers in many countries that are developing use polluted or untreated water for irrigation and untreated or polluted human or animal dung as fertilizer, which contributes to increased transmission of intestinal parasitic (IP) contaminations and is to blame for the high rates of contamination with parasites [4]. Fruits and vegetables can be contaminated by protozoans (cysts and oocytes) and helminths (eggs and larvae), and humans get infected by consuming those contaminated fruits and vegetables without proper washing [5]. Such contamination is high in developing countries like Ethiopia due to poverty, low level of environmental sanitation, and ignorance of simple health promotion [6].

According to a study conducted in different parts of the country, the prevalence of intestinal parasite is high in the country's population, particularly in those who consume raw fruits and vegetables [6–10]. Between 25.1% and 57.8% of the fruit and vegetable samples gathered throughout the country's marketing phase were thought to be parasite-contaminated [6, 10–12].

Fruits and vegetables are frequently seen being sold in Bule Hora Town's open markets and on the streets, where they are being eaten by inhabitants. Although the town of Bule Hora has a high prevalence of intestinal parasitosis [13], data on the prevalence of intestinal parasites on raw fruits and vegetables are scarce. Therefore, the purpose of this study was to assess the prevalence of intestinal parasites and its associated factors among fruits and vegetables collected from local markets of Bule Hora Town, Southeast Ethiopia.

## 2. Methods

### 2.1. Study Area and Study Period

This research on vegetables and fruits was done in Bule Hora Town from July 29 to August 17, 2022. Bule Hora Town is found on the asphalt Addis Ababa-Moyale Road in the West Guji Zone of the Oromia Region, 476 kilometers from the country's capital, 109 kilometers south of Dilla, and 302 kilometers north of Moyale. It is located at an altitude of 1,716 meters above sea level and has coordinates of 5°35′N and 38°15′E, respectively. The town has 27,820 populations, where 14,519 males and 13,301 females were recorded as living in Bule Hora Town overall in the 2014 National Census [13]. Fruits and vegetables are frequently seen sold in Bule Hora Town's open markets, on the streets, and in overcrowded areas. In addition, the vendor did not use the table or any material to put the produce away from dirt.

### 2.2. Study Design and Population

A cross-sectional study was conducted on selected raw fruits and vegetables sold in selected local markets in Bule Hora Town to assess the prevalence of intestinal parasites on raw fruits and vegetables collected from selected local markets in Bule Hora Town.

### 2.3. Inclusion and Exclusion Criteria

The study included those freshly purchased fruits and vegetables. Spoiled fruits and vegetables were excluded from the study.

#### 2.3.1. Sample Size

The 36.3% proportion was used to calculate the sample size using a single population proportion formula from a study conducted in the Butajira Town of the Gurage Zone, Southern Ethiopia [7], with a confidence level of 95% and a degree of precision 5%. Accordingly, the calculated sample size was 355. Considering 10% of the nonresponding rate, the final sample size was **391**.

#### 2.3.2. Sampling Technique

**Figure 1:**
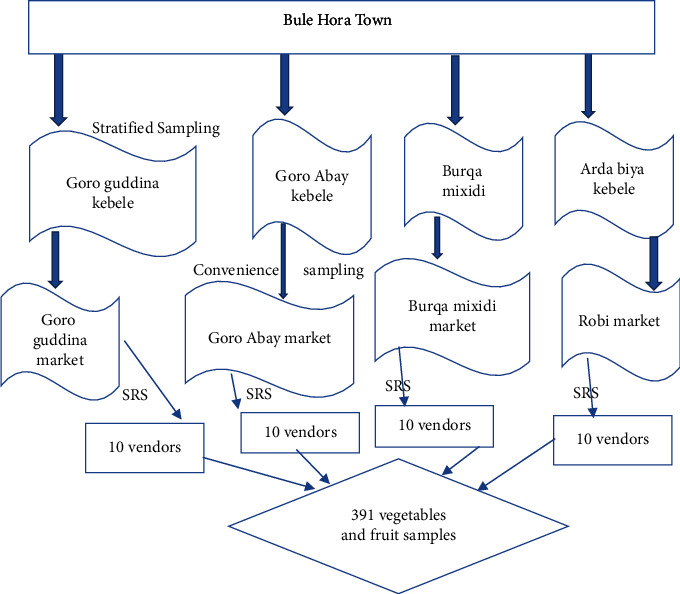
Sampling technique for the assessment of intestinal parasites and its associated factors among fruits and vegetables collected from local markets of Bule Hora Town, Southeast Ethiopia.

A multistage sampling technique was used. First, we selected four kebeles (the smallest administrative unit in Ethiopia) from the town conveniently. Then, a stratified sampling technique was used to classify the local market in each selected kebele into different strata. To draw fruit and vegetable vendors from each local market, simple random sampling (SRS) was used. Finally, about 391 samples of vegetables and fruits were collected from all selected vendors (Figure 1).

### 2.4. Data Collection Methods

#### 2.4.1. Data Collection

Three trained data collectors gathered the sample and conducted the interviews. Data on the variables (sex of vendors, vendor status of handwashing with soap after using toilet, vendor status of eating raw vegetables or fruits, produce type, and washing vegetables or fruits before display) connected to the presence of parasites on fruits and vegetables were gathered using a pretested structured questionnaire. After data gathering, the local language data were translated back into English with the assistance of experts. The simple observation was used to record information on the market's type and display methods.

#### 2.4.2. Laboratory Processing

Samples were obtained from merchants who were chosen randomly in each market under the same circumstances as regular purchases. This survey included participation from 40 vendors in total. Eight types of fruits and vegetables such as *Mangifera indica* (mango), *Brassica oleracea* (cabbage), *Daucus carota* (carrot), *Lycopersicon esculentum* (tomato), *Capsicum annuum* (green pepper), *Persea americana* (avocado), *Citrus sinensis* (orange), and *Spinacia oleracea* (spinach) were collected from four selected local markets during the data collection period. Each sample was appropriately labeled and placed in plastic bags before being transported to Bule Hora University's Medical Parasitology Laboratory for parasitological analysis.

#### 2.4.3. Microscopic Examination

To detect the stages of helminths and protozoan parasites that are typically thought to be linked to the contamination of ova, larvae, cysts, and oocysts, a portion (200 g) of each fruit and vegetable was washed separately in 500 ml of normal saline (0.85% NaCl). To allow for appropriate sedimentation, the washing solution was then let to stand on the bench for a whole night. Then, using a Pasteur pipette to collect the supernatant, 15 ml of the sediment was transferred to a centrifuge tube using a sieve to filter out unwanted components. The tube was centrifuged for five minutes at 3000 rpm to concentrate the parasitic stages [7].

After centrifugation, the sediment was gently stirred by hand to redistribute the parasitic stages before the supernatant was decanted carefully without shaking. The 100 *μ*l sediment was then placed on a clean glass slide and covered with a cover slide before being examined using a light microscope with a 10× and 40× objective lens. In addition, using the standard protocol previously published [11, 14, 15], the modified Zeihl–Neelsen staining technique was utilized to identify coccidian protozoan oocysts such as *Cryptosporidium* spp., *Isospora belli*, and *Cyclospora cayetanensis*.

As a result, a thin smear was made immediately from the silt and let to dry naturally. Each slide was then treated with methanol 5 minutes later and stained with carbol fuchsin 30 minutes later. After staining, the slide was rinsed with tap water and decolored for 1–3 minutes with acid alcohol. The slides were then counterstained with methylene blue for one minute after being cleaned with tap water. The slide was then rinsed with tap water and let air dry. The slide was then examined using 100× objectives on a light microscope.

#### 2.4.4. Quality Assurance

The questionnaire was first prepared in English and then translated into the local language (*Amharic* and *Afaan Oromo*) by consulting language experts. Questionnaires were checked regularly for completeness of required data. The training was given to the data collector before data collection. The sample was prepared and examined properly. Data were checked by principal investigators and supervisor for completeness and accuracy of data collection daily. The laboratory result recording form was kept properly for checking.

#### 2.4.5. Data Analysis

Data were cleaned and processed using SPSS for Windows version 25 after being coded, input, and cleaned into EpiData version 3.1. For the analysis of the data, inferential as well as descriptive statistics were used. To describe the study population with the relevant variables, frequencies and summary statistics including means, standard deviations, and percentages were generated. Using the binary logistic regression analysis, the association and significance between the dependent and independent variables were assessed. To control confounding variables, a multivariate analysis was also performed using the backward stepwise approach. *P* values and the 95% confidence interval (CI) for the odds ratio (OR) were used in the studies to determine the significance of the associations. Therefore, a *P* value less than 0.05 at 95% CI was considered significant.

## 3. Ethical Approval

The Bule Hora University Institute of Health provided an ethical approval letter, while the Bule Hora Zonal Health Department, Bule Hora Zonal Police Department, and the Zonal Trade Department each provided an official permission letter. Before collecting data, the respondents (vendors of fruits and vegetables) were informed of the study's objectives, and verbal consent was acquired. The Institute of Health at Bule Hora University approved the verbal consent.

## 4. Results

### 4.1. Frequency Distribution of Parasitological Contamination of Fruits and Vegetables

In this study, 142 samples were identified as being contaminated with at least one type of parasite; the overall contamination rate was 36.3%. Of the contaminated fruits and vegetables, 130 (91.5%) had single contamination and 12 (8.5%) had mixed contamination. Of each sample group collected, cabbage was the most contaminated 27 (7%) product followed by tomato 25 (6.4%), green pepper 23 (5.9%), carrot 22 (4.8%), spinach 19 (4.9%), banana 14 (3.6%), mango 12 (3.1%), and avocado 11 (2.8%) (Table 1).

### 4.2. Intestinal Parasitic Contamination of Vegetables and Fruits

Of 391 samples of fruits and vegetables assessed, 142 (36.3%) were positive for various intestinal parasites. About seven different intestinal parasites were identified. The predominant parasite detected was *Ascaris lumbricoides* 48 (12.3%), followed by *Entamoeba histolytica/dispar* 41 (10.5%), *Giardia lamblia* 25 (6.4%), *Trichuris trichiura* 12 (3.1%), hookworm 12 (3.1%), *Enterobius vermicularis* 2 (0.5%), and *Taenia* spp. 2 (0.5%). No intestinal coccidian were found (Table 2).

### 4.3. Factors Associated with Parasitic Contamination of Fruits and Vegetables

Most independently measured variables do not fit the bivariate model. With a *P* value less than 0.25, about eight variables were candidates for multiple logistic regressions. Finally, using multiple logistic regression models, four variables indicate significant associations with *P* values less than 0.05. Produce type, handwashing with soap after using toilet, eating raw vegetables or fruits, and washing fruits or vegetables before display were variables substantially associated with the prevalence of intestinal parasites.

Produce type was independently associated with the intestinal parasitic contamination (AOR = 2.3; 95% CI: 1.35–3.76). According to these findings, vegetables were approximately two times more likely to be contaminated compared to fruits. Handwashing with soap after using toilet was another factor independently associated with the contamination of vegetables and fruits (AOR = 3.043; CI: 1.834–5.047). When compared to those who wash their hands with soap after using the toilet, fruits and vegetables of vendors who does not wash their hands with soap after the toilet were around three times more likely to get intestinal parasitic contamination.

Besides, washing fruits and vegetables before the display is associated with intestinal parasitic contamination (AOR = 5.585; 95% CI: 3.370–9.255). When compared to produce that was washed before presentation, the likelihood of it being contaminated with at least one parasite was 5 times higher. Eating raw fruits or vegetables is another factor that is associated with intestinal parasitic contamination (AOR = 0.604; 95% CI: 0.394–0.925) (Table 3).

## 5. Discussion

Due to the favorable environment and unhygienic conditions that encourage fecal pollution of water, food, and soil, intestinal parasites are extensively dispersed in Ethiopia, like in many tropical nations [16]. Fruits and vegetables may become contaminated with parasitic pathogens as a result of the market chain, where they are likely to travel through multiple hands [16–18]. The goal of the current study was to determine the prevalence and level of contamination of several intestinal parasites in various fruits and vegetables sold in Bule Hora Town's local markets in Southern Ethiopia. In the present study, a 36.3% parasitic contamination rate was discovered, which is consistent with studies from Thailand [19], the Gurage Zone [9], and Bahir Dar City [7]. However, the parasitic contamination rate in from the current studies is higher than a study reported from Egypt [20], the United Arab Emirates [21], and Arba Minch Town [6]. On the other hand, our finding has a lower parasitic contamination rate than studies from Jimma Town [11], Dire Dawa [10], and Dawuro Zone[16]. The discrepancy between this study and others could be related to variances in the sample size, inadequate postharvest, socioeconomic level, and geographic variations.

In these studies, cabbage (7%) and tomato (6.4%) were found to be the most frequently contaminated product followed by green pepper 23 (5.9%), carrot 22 (4.8%), spinach 19 (4.9%), banana 14 (3.6%), mango 12 (3.1%), and avocado 11 (2.8%). This finding is in line with a study conducted in Jimma [11], Bahir Dar City [7], Gurage Zone [9], and Eastern Showa [22]. There is a variation in contamination between the vegetables and fruits in the abovementioned studies with mostly higher contamination rates reported on vegetables relative to fruit. This might be due to the difference in processing, handling, and exposure to the surface which may facilitate the parasites to attach to the product.

With a prevalence of 12.3%, *Ascaris lumbricoides* was the parasite that was found most frequently in this study. The prevalence of *Ascaris lumbricoides* is consistent with research carried out in Kenya and the Philippines [11, 20]. This dominance may be related to the widespread distribution of the parasite, the large number of eggs laid by the fecund female parasite, which also contributes to the widespread distribution of the parasite, and the robust and resistant nature of the eggs, which allows them to survive unfavorable conditions. The eggs can survive without oxygen, endure desiccation for two to three weeks, and remain viable for two years at a temperature of 5– 10°C [11, 23].

In general, compared to other similar studies conducted in different areas, a higher rate of parasitic contamination of fruits and vegetables was observed in this study. This could be attributed to a variety of factors, including geographic location, the type and number of samples examined, the methods used to detect intestinal parasites, the type of water used for irrigation, and postharvest handling techniques, which vary from one country to another. Individual hygiene practices, sanitary facilities, and climatic conditions all significantly influence outcomes in addition to the aforementioned determinants [11, 23].

Regarding factors-assessed odds of unwashed produce before display becoming contaminated with at least one parasite was more than 5 times (AOR = 5.585; 95% CI: 3.370–9.255) when compared to those washed before display. This finding was in line with the study in Bahir Dar City [7], Jimma Town [11], and Arba Minch Town [6]. This might be because washing the produce before display helps to reduce the parasites. However, this finding disagrees with the study in the Gurage Zone [9]. The discrepancy might be due to the quality of water used for washing the produce.

Produce type was another variable independently associated with the intestinal parasitic contaminations (AOR = 2.3; 95% CI: 1.35–3.76). According to these findings, vegetables were approximately two times more likely to get contaminated compared to fruits. This finding was in line with a study in Bahir Dar City [7]. This might be due to the risk of contamination of the products in the field, during transportation, and other postharvest-related activities [11, 23].

Eating raw fruits or vegetables is another factor that is linked to intestinal parasitic contamination (AOR = 0.604; 95% CI: 0.394–0.925). According to this finding, vendors who do not eat raw vegetables or fruits have about 60% less probability of contaminating vegetables and fruits compared to those vendors who eat raw vegetables or fruits. This might be due to the consumption of raw vegetables and fruits being linked to intestinal parasitic contamination for those vendors, and they may contaminate the produce they are vending during handling.

## 6. Conclusion

This finding showed high contamination of fruits and vegetables consumed in the study area by intestinal parasites. The authors contend that adequate vegetable washing, improved sanitary practices for those who handle vegetables, and improvements in sanitation standards can all help prevent contamination, which is still the most efficient strategy to reduce parasitic illnesses that are spread by fruits and vegetables. Farmers and vendors should also receive thorough health education. When displaying produce for sale, vendors should make sure it stays off the ground. Further study is required to determine the extent to which farm products, water, and the soil where fruits and vegetables are grown are contaminated by parasites.

## Figures and Tables

**Table 1:** Frequency distribution of parasitological contamination of fruits and vegetables sold in local markets of Bule Hora Town, Southern Ethiopia, from July 29 to August 17, 2022.

Types of vegetables and fruits	Number examined	Number positive (%)	Number of parasitic species detected (%)		
			One	Two	Three
Cabbage	59	27 (7)	14	2	0
Carrot	56	22 (5.6)	17	1	1
Spinach	48	19 (4.9)	15	0	0
Banana	37	14 (3.6)	15	1	0
Mango	37	12 (3.1)	14	0	0
Avocado	39	1 1 (2.8)	19	1	1
Green pepper	56	23 (5.9)	18	2	0
Tomato	59	25 (6.4)	18	2	1
Total	391 (100%)	142 (36.4)	130 (33.2)	9 (2.4%)	3 (0.8%)

**Table 2:** Assessment of intestinal parasites and its associated factors among fruits and vegetables sold in local markets of Bule Hora Town, Southern Ethiopia, from July 29 to August 17, 2022.

Detected parasite	Frequency	Prevalence (%)
*Entamoeba histolytica/dispar*	41	10.5
*Giardia lamblia*	25	6.4
Hookworm	12	3.1
*Ascaris lumbricoides*	48	12.3
*Enterobius vermicularis*	2	0.5
*Trichuris trichiura*	12	3.1
*Taenia* spp.	2	0.5
Total (positive)	142	36.3
Negative	249	63.6

**Table 3:** Binary logistic regression tables for factors associated with parasitic contamination of fruits and vegetables sold in Bule Hora Town, South East Ethiopia, 2022 (*n* = 391).

Independent variables		Presence of intestinal parasite		COR (95% CI)	AOR (95% CI)
		Negative (%)	Positive (%)		
Sex of vendors	Male	105 (70%)	45 (30%)	1	
	Female	144 (59.7%)	97 (40.3)	0.636 (0.412–0.982)	

Vendors' status of eating raw vegetables or fruits	Yes	116 (70.3%)	49 (29.7%)	0.604 (0.394–0.925)	0.604 (0.394–0.925)^*∗*^
	No	133 (58.8%)	93 (41.2%)	1	1

Vendors' status of handwashing with soap after using toilet	Yes	92 (70.2%)	39 (29.8%)	1	1
	No	54 (41.9%)	75 (58.1%)	1.99 (1.274–3.108)	3.043 (1.834–5.047)^*∗*^

Vendors' status of fingernail trimming	Yes	142 (57.9%)	103 (42.1%)	1	
	No	107 (73.3%)	39 (26.7%)	1.99 (1.274–3.108)	

Washing fruits and vegetables before display	Yes	159	39	1	
	No	90	103	1.873 (1.234–2.842)	5.585 (3.370–9.255)^*∗*^

Produce type	Fruits	113	41	1	1
	Vegetables	136	101	2.047 (1.318–3.179)	2.253 (1.351–3.758)^*∗*^

Statistical significance at *P* < 0.05 = ^*∗*^, COR = crude OR, AOR = adjusted OR, and CI = confidence interval.

## Data Availability

All required datasets on which the conclusion of the paper depends on are included within the manuscript.
